# Clinical Trial: A Pragmatic Randomised Controlled Study to Assess the Effectiveness of Two Patient Management Strategies in Mild to Moderate Ulcerative Colitis—The OPTIMISE Study

**DOI:** 10.3390/jcm13175147

**Published:** 2024-08-30

**Authors:** Silvio Danese, Gionata Fiorino, Eric Vicaut, Kristine Paridaens, Asiya Ugur, Brian Clark, Tomas Vanasek, David Stepek, Ferdinando D’Amico, Rachel West, Lennard P. L. Gilissen, Maria Wisniewska Jarosinka, Piotr Drobinski, Grzegorz Fronik, Mirosław Fic, Michał Walczak, Maciej Kowalski, Bartosz Korczowski, Michal Wiatr, Laurent Peyrin-Biroulet

**Affiliations:** 1 Department of Gastroenterology and Digestive Endoscopy, San Raffaele Hospital, Vita-Salute San Raffaele University, 20132 Milan, Italy; damico_ferdinando@libero.it; 2 IBD Unit, Department of Gastroenterology and Digestive Endoscopy, San Camillo-Forlanini Hospital, 00152 Rome, Italy; gionataf@gmail.com; 3 Clinical Trial Unit, Hospital Lariboisière APHP, 75010 Paris, France; eric.vicaut@aphp.fr; 4 Ferring International Center S.A, 1162 Saint-Prex, Switzerland; kristine.paridaens@ferring.com; 5 Ferring Pharmaceuticals A/S, 2770 Kastrup, Denmark; asiya.ugur@ferring.com (A.U.); brian.clark@ferring.com (B.C.); 6 Hepato-Gastroenterologie HK, s.r.o., 50012 Hradec Králové, Czech Republic; tomas.vanasek@hepato-gastro.com; 7 Internal Department, Military Hospital Brno, 61500 Brno, Czech Republic; dstepek@vnbrno.cz; 8 Department of Gastroenterology and Hepatology, Franciscus Gasthuis & Vlietland, 3045 PM Rotterdam, The Netherlands; r.west@franciscus.nl; 9 Department of Gastroenterology and Hepatology, Catharina Hospital Eindhoven, 5623 EJ Eindhoven, The Netherlands; lennard.gilissen@catharinaziekenhuis.nl; 10 Department of Gastroenterology, Medical University of Lodz, 92-213 Lodz, Poland; majkawj@bmp.net.pl; 11 Centrum Medyczne Lukamed in Chojnice, 89-600 Chojnice, Poland; piotrdrobinski@lukamed.com; 12 Melita Medical—Centrum Proktologii, Onkologii i Chorób Jelit, 50-449 Wrocław, Poland; g.fronik@melitamedical.pl; 13 Polish Society of Gastroenterology, 58-521 Jezów Sudecki, Poland; m.fic@kcbk.pl; 14 Institute of Human Genetics, Polish Academy of Sciences, 61-772 Poznan, Poland; mi.walczak@wp.pl; 15 Department of Gastroenterology, Centrum Diagnostyczno—Lecznicze Barska, 87-806 Włocławek, Poland; makabi@wp.pl; 16 Institute of Medical Sciences, Medical College, University of Rzeszów, 35-310 Rzeszów, Poland; korczowski@op.pl; 17 Ośrodek Badań Klinicznych CLINSANTE S.C. Ewa Galczak-Nowak Małgorzata Trzaska, Chałubińskiego, 85-004 Bydgoszcz, Poland; michalwiatr2019@gmail.com; 18 Department of Gastroenterology and Inserm NGERE U1256, University Hospital of Nancy, University of Lorraine, 54000 Vandoeuvre-lès-Nancy, France; peyrinbiroulet@gmail.com; 19 Department of Gastroenterology, Nancy University Hospital, 54500 Vandœuvre-lès-Nancy, France; 20 INFINY Institute, Nancy University Hospital, 54500 Vandœuvre-lès-Nancy, France; 21 FHU-CURE, Nancy University Hospital, 54500 Vandœuvre-lès-Nancy, France; 22 Groupe Hospitalier Privé Ambroise Paré—Hartmann, Paris IBD Center, 92200 Neuilly sur Seine, France; 23 Division of Gastroenterology and Hepatology, McGill University Health Centre, Montreal, QC H4A 3J1, Canada

**Keywords:** ulcerative colitis, treat-to-target, faecal calprotectin, inflammatory bowel disease, tight monitoring

## Abstract

**Background:** Current management of mild-to-moderate ulcerative colitis (UC) involves monitoring clinical markers of disease activity, such as stool frequency (SF) and rectal bleeding (RB), and adjusting treatment accordingly. Our aim was to assess whether targeting treatment based on faecal calprotectin (FC) levels (treat-to-target; T2T) provides greater UC disease control versus a symptom-based approach. **Methods:** This was a pragmatic, randomised (1:1) controlled study of patients with mild-to-moderate UC (global Mayo score 2–6) treated with ≤2.4 g/day 5-aminosalicylic acid that compared the effectiveness of two management strategies with (interventional arm) and without (reference arm) FC home monitoring over 12 months of follow-up. Treatment was optimised in the interventional arm using FC values and clinical symptoms (PRO-2), while the reference arm used only PRO-2. **Results:** 193 patients completed the study. No significant difference was found for the primary endpoint (Mayo Endoscopic Subscore [MES] = 0 at 12 months). A numerical advantage for the interventional arm over the reference arm for the primary endpoint (37.0% vs. 33.4%, respectively) and for MES ≤ 1, RB = 0, and SF ≤ 1 at 12 months was found following imputation for missing data. The composite endpoint of MES = 0, RB = 0, and SF ≤ 1 at 12 months was achieved at a significantly higher rate in the interventional arm than the reference arm (effect size [ES]: 0.17, 95% CI 0.02–0.32; *p* < 0.05). A similar result was obtained for MES ≤ 1, RB = 0 and SF ≤ 1 (ES: 0.22; 95% CI 0.07–0.37; *p* < 0.05). **Conclusions:** T2T using FC monitoring was effective in patients with mild-to-moderate UC at 12 months. Further longer-term studies are required to confirm the results.

## 1. Introduction

Ulcerative colitis (UC) is a chronic, inflammatory condition of the rectum and colon of unknown aetiology [1,2]. Up to 75% of patients follow a mild-to-moderate disease course, whereas up to 25% may need therapy escalation and, in the case of failure of all medications, may undergo proctocolectomy [3,4,5,6,7,8]. The European Crohn’s and Colitis Organisation’s (ECCO) guidelines on the medical management of UC recommend a step-up approach for mild-to-moderate UC [4,9]. Usually, 5-aminosalicylic acid (5-ASA) compounds, orally and rectally, are the first-line therapy for patients with mild-to-moderate flares, followed by second generation corticosteroids, systemic steroids, immunomodulators, biological agents, and small molecules in a stepwise approach in case of non-response or intolerance [9]. However, there is no clear evidence on timely escalation and de-escalation of therapies and what the best targets are to be used as a basis for clinical decisions in these patients. In 2014, the Selecting Therapeutic Targets in Inflammatory Bowel Disease (STRIDE) Consensus agreed on the main targets for therapy [10]. In patients with UC, they recommended resolution of clinical signs of disease activity and endoscopic remission as the optimal targets. Remission of intestinal inflammation biomarkers, such as C-reactive protein (CRP) and faecal calprotectin (FC), was considered as an adjunctive target due to a lack of scientific evidence to recommend treatment optimisation by taking into account such levels alone. More recently, the STRIDE-II Consensus recommended symptomatic relief and normalisation of serum and faecal markers as short-term targets for all therapies [11]. FC is an antimicrobial substance released by activated or damaged polymorphonuclear neutrophilic granulocytes (PMN) and other cells, such as monocytes, macrophages, and epithelial cells. The concentration of calprotectin in faeces is correlated with the number of PMNs migrating into the gut lumen, and it can be detected reliably even in small (less than one gramme) random stool samples [12,13]. Several studies have shown the good predictive value of FC both for endoscopic remission and histological healing [14]. Many patients with inflammatory bowel disease (IBD) in clinical remission with normal CRP levels still have active on-going colonic inflammation, reflected by increased FC. Such patients have an increased risk of relapse within a few months [15,16]. Since mucosal healing is associated with a reduced risk of relapse and the need for expensive medical treatment and/or major abdominal surgery, targeting colonic inflammation despite the absence of symptoms or systemic inflammation may be clinically appropriate [17,18,19,20,21]. The results from the CALM trial in Crohn’s disease (CD) showed that adjusting treatments based on FC levels instead of symptoms alone might engender a significantly higher rate of endoscopic remission [22]. At present, no similar studies are published in mild-to-moderate UC. There are no studies demonstrating that management based on symptom control and normalisation of faecal calprotectin in UC is superior to symptom control alone. We aimed to investigate whether a treat-to-target (T2T) approach based on monitoring of non-invasive parameters, such as clinical symptoms and FC, can provide a significantly higher benefit for patients with mild-to-moderate UC versus an entirely symptom-based approach in terms of disease control and quality of life.

## 2. Materials and Methods

This was a prospective, pragmatic, randomised, controlled, open-label, multicentre study.

### 2.1. Study Objectives and Endpoints

The primary objective was to assess and compare the effectiveness of two patient management strategies in obtaining mucosal healing at 12 months. Secondary objectives were to assess and compare the effectiveness of the two strategies at 12 months in regard to: absence of rectal bleeding (RB); normalisation of stool frequency (SF); endoscopic remission; improvement in quality of life (QoL) and work productivity; and need for rescue therapy (systemic corticosteroids, immunosuppressants, biologics, or small molecules). The primary endpoint of mucosal healing was defined and measured as the percentage of patients with Mayo endoscopic subscore (MES) = 0 at 12 months. Secondary endpoints were defined and measured at month 12 as: percentage of patients with RB = 0; percentage of patients with SF ≤ 1; percentage of patients with MES ≤ 1 (endoscopic remission); percentage of patients escalated to rescue therapy; and any score changes of the Short IBD Questionnaire (SIBDQ), Short Form-36 (SF-36), and Work Productivity and Activity Impairment (WPAI) questionnaires. Only in the interventional arm were FC value changes at study timepoints assessed.

### 2.2. Study Population

All consecutive adult patients (≥18 years old) with active mild-to-moderate UC, defined with a total Mayo score between 2 and 6, with RB ≥ 1 and MES ≥ 1, were considered eligible to participate in the study. Other inclusion criteria were use of oral 5-ASA ≤ 2.4 g/day or no treatment at the time of inclusion, ability to use the FC Home Test, access to the internet and a smartphone with camera, and ability to understand and comply with all the study procedures. Patients with contraindications to 5-ASA and/or second generation corticosteroids as per the relevant Summary of Product Characteristics (SmPC), currently on a nonsteroidal anti-inflammatory drug (NSAID) or proton-pump inhibitor (PPI), exposed to immunomodulators and/or biologics within 6 months prior to baseline, with positive stool culture up to 2-weeks prior to baseline, pregnant orbreastfeeding women, known mental incapacity or language barriers precluding adequate understanding of the Informed Consent information and the study activities, or having any other reasons to be unable to participate based on the judgement of the investigator, were excluded.

### 2.3. Interventions

During the screening phase, all patients were first investigated for infections, including exclusion of Clostridium difficile infection, as recommended by the ECCO Guidelines. When the new UC flare was confirmed, patients were randomised 1:1 (by central randomisation with a computer-generated list) to one of the following study arms:

- Interventional arm: monthly monitoring during the active phase of the disease, and quarterly during remission. UC treatment optimisation (escalation/de-escalation) was performed by the investigator and triggered based on patient self monitoring of FC values and/or Patient Reported Outcome-2 (PRO2) scoring.
- Reference arm: quarterly monitoring during the active phase of the disease, and every 6 months during remission. UC treatment optimisation (escalation/de-escalation) was performed by the investigator and triggered based on PRO-2 scoring only.

At randomisation (Day 0), all patients were prescribed rectal 5-ASA (1 g/day) plus oral 5-ASA ≥ 2.4 g/day as first-line therapy, in accordance with current ECCO Guidelines for the management of mild-to-moderate UC.4 In addition, patients completed the SIBDQ, SF-36, and WPAI questionnaires. Patients completed the PRO-2 before seeing the study doctor or nurse in order to not influence the responses. To minimise response variability (e.g., due to response fatigue), the recommended order to administer the questionnaires was SIBDQ, SF-36, and WPAI. Questionnaires were then assessed and reviewed by the study investigator with the purpose of reporting any adverse event (AE) noted by the patient. All questionnaires were repeated at months 6 and 12 in both arms. Patients also received an e-diary to be completed at home within 1-week prior to each scheduled visit/call (patients received automated alerts via the e-diary portal) or on-demand when they felt the need. The e-diary recorded information about PRO-2 clinical symptoms (RB and SF) within the last 3 days.

**Figure 1:**
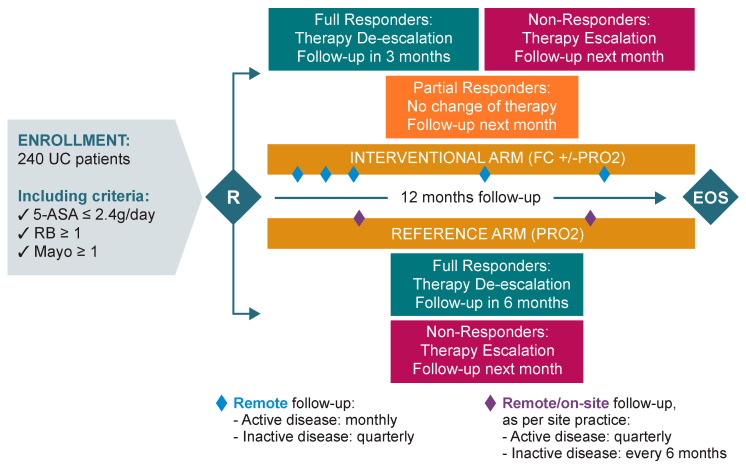
Study design. QoL: Quality of life; HCP: Health care professional; PRO: Patient-reported outcome; RB: Rectal bleeding; UC: Ulcerative colitis; FC: Faecal calprotectin.

Depending on the study arm, follow-up procedures were planned as follows (Figure 1).

For the interventional arm, the investigator/designee provided the patient with CalproSmart™ FC Home tests (Calpro AS, Arnstein Arnebergs vei 30, 1366 Lysaker, Norway) to be used for the whole duration of the study, along with all the necessary instruction materials. One demo test was used to educate the patient during the baseline on-site visit. Study investigators also helped the patient register the CalproSmart™ application. The patients were then asked to perform the next test at home within the following 24 h to ensure that they remembered how to perform the test and to solidify their knowledge. Upon reception of a CalproSmart™ portal alert, investigators recorded the FC value in the patient medical record and in the electronic Case Report Form (eCRF). Subsequently, patients received alerts 7 days prior to each scheduled monthly/quarterly phone call to perform the FC home test. Patients performed the FC test (preferably in the morning) at home, and the results were automatically sent from the patient’s smartphone to the CalproSmart™ portal, from where it was viewed by the investigator/designee. Similarly, the patient received alerts 7 days prior to each scheduled monthly/quarterly phone call to complete their clinical symptoms (PRO-2) through the e-diary.

Follow-up was done remotely via phone calls, at a monthly frequency if the disease was still active (and until remission was achieved), or on a quarterly frequency if the disease was in remission. In the case of a relapse of symptoms, monthly monitoring calls were restarted until remission was achieved, or until the end of the study (whichever came first). During the course of the study, follow-up visits (and any other appropriate assessments) could be done on site if judged necessary by the doctor and/or as per site practice.

The investigator assessed both the FC value and PRO-2 scoring reported at the defined timepoints and optimised the UC therapy and monitoring as follows:

- If patients were non-responders (defined as FC ≥ 150 μg/g, irrespective of PRO-2 scoring), therapy was escalated to the next escalation step (Table 1). If current therapy administered had not reached the minimum duration of therapy as per the SmPC, the investigator could decide to continue the current therapy or to escalate, in the best interest of the patient. In all cases, the next follow-up call was scheduled for the following month.

- If patients were partial responders (defined as FC < 150 μg/g and PRO-2 ≥ 2), the investigator maintained the current therapy administered if the SmPC allowed (or de-escalated to oral 5-ASA ≥ 2.4 g/day if not). The next follow-up call was scheduled for the following month.
- If patients were full responders (defined as FC < 150 μg/g and PRO-2 ≤ 1, with RB = 0), the investigator first de-escalated the current therapy to oral 5-ASA ≥ 2.4 g/day. De-escalation started only once current therapy administered had reached the minimum duration of therapy as per the SmPC. The next follow-up call was scheduled in 3 months. At the next visit, therapy was managed as follows:
  1. For full responders, the investigator proceeded to the second de-escalation step/maintenance therapy of 5-ASA ≤ 2.4 g/day and the next follow-up call was scheduled in 3 months.
  2. For partial responders, the investigator maintained the therapy unchanged and scheduled the next follow-up call in 1 month.
  3. For non-responders, the investigator escalated the therapy to the next level and scheduled the next follow-up call in 1 month.

If the investigator suspected a transitory increase in FC levels (e.g., if the patient had taken NSAIDs or had an upper respiratory tract infection with mucus), the investigator could request to repeat the FC test in 2–4 weeks to confirm if the FC value was ≥150 μg/g, before adjusting therapy. In all cases, if FC test results showed two consecutive FC values ≥150 μg/g, the investigator was to escalate therapy. In addition, whenever the patient felt the need, i.e., in the case of a flare of symptoms (increased SF and/or RB) while he/she was in remission, they were allowed to perform the FC test at home in between scheduled calls. The patient then sent the FC value to the investigator via the CalproSmart™ portal and was then contacted to adjust their therapy as needed.

For the reference arm, the first follow-up visit was done 3 months post baseline. If the PRO-2 assessment showed that the patient was still a non-responder to current therapy, monitoring was continued on a quarterly basis until remission was achieved. If the patient responded to therapy, monitoring was done on a 6 monthly basis. If a relapse occurred during the course of the study, quarterly monitoring was restarted until remission was achieved or until the end of the study (whichever came first).

During each follow-up visit, the investigator reviewed the PRO-2 assessment via the e-Diary web portal. Predicated on the PRO-2 scoring reported, the investigator optimised the patient’s UC therapy and monitoring as follows:

- For non-responders (defined as PRO-2 ≥ 2), the investigator escalated to the next step (Table 1) and scheduled the next follow-up visit in 3 months.
- For full responders (defined as PRO-2 ≤ 1 with RB = 0), the investigator first de-escalated therapy to oral 5-ASA ≥ 2.4 g/day (waiting for the time indicated in the SmPC), and the next follow-up visit was scheduled in 6 months. If the patient was still a full responder during the following visit, the investigator proceeded to the second de-escalation step/maintenance therapy of 5-ASA ≤ 2.4 g/day, and the next follow-up visit was scheduled in 6 months, or at the end of the study visit, whichever came first. Whereas if the patient was a non-responder at the following study visit, therapy was escalated to the next level, and the subsequent follow-up visit was scheduled in 3 months.

Irrespective of the study arm, the medical management (therapy optimisation) was the same for all enrolled patients and prescribed by the treating doctor in line with the current ECCO Guidelines and the SmPC recommendations. All patients were monitored for a total study duration of 12 months, and at that timepoint, all underwent a final clinical and endoscopic assessment.

All the clinicians involved in the study participated in person or virtually in preliminary investigator meetings to standardise the monitoring strategies in the interventional group.

### 2.4. Statistical Analysis

#### 2.4.1. Sample Size

Assuming a success rate of 30% in the reference arm and 50% in the interventional arm, it was calculated that 206 patients were needed to provide a power of 80% to detect a difference of 20% between the study groups, with an alpha = 0.05, two-sided; 1:1 allocation. The sample size was increased by 15% to 240 to account for possible drop-outs.

#### 2.4.2. Effectiveness Analysis Set

Effectiveness endpoints were analysed in the modified intention-to-treat (mITT) population, which included all randomised patients except those with entry criteria violations, and by per-protocol (PP) analysis, which included all patients included in the mITT population except those with major protocol deviations.

#### 2.4.3. Statistical Tests

For continuous data, descriptive statistics (N, mean, standard deviation, median, interquartile range, minimum and maximum values) were performed. For categorical data, tabulations of N (%) were done. Missing values after randomisation for primary and secondary effectiveness endpoints were imputed using non-responder imputation. Data for patients who discontinued the study or who moved to a rescue therapy outside the scheduled escalation/de-escalation steps (Table 1) and/or due to a severe flare of the disease were also subject to non-responder imputation. Chi-square, two-sample Wilcoxon rank-sum test, and Mann–Whitney tests were performed depending on the nature and distribution of the data. For the primary endpoint, comparing the percentage of patients achieving MES = 0 at 12 months, the Chi-square test was used.

Due to the recognised impact of the COVID-19 pandemic on enrolment and participation, a complementary analysis was undertaken to account for missing data in the mITT population. First, the patients with entry criteria violations were reassessed to ensure that only those with a clinical rationale for exclusion (i.e., those with violations related to baseline medications, or those who had severe disease activity at baseline) were removed from the mITT population, thereby enabling more data to be available for analysis. Second, Monte Carlo Markov Chain (MCMC) multiple imputation was used to impute missing values based on analyses of the relationships between baseline and postbaseline data and outcomes for the patients with complete data. Following MCMC imputation, the primary (MES = 0) and secondary outcomes for MES ≤ 1, RB = 0, and SF ≤ 1 were reanalysed. Logistic regression analysis was also performed by mapping the initial MCMC results to a theoretical population of 250 patients, with planned treatment (interventional arm or reference arm) used as the dependent variable and 12 month results for MES, SF, and RB used as the predictor variables. The derived predictor function was then used for receiver operating characteristic (ROC) curve analysis against the planned treatment. To check the significance of the result, correlation (Kendall’s Tau and Spearman’s rho) and Mann Whitney tests were performed, using planned treatment as the grouping variable and the logistic regression predictor function as the test variable. A meta-analysis was subsequently undertaken using the MCMC results for MES = 0 or MES ≤ 1, RB = 0 and SF ≤ 1 transcribed to a population size matching that of the mITT population. Effect sizes (ES) were calculated together with the means and standard errors.

For hypothesis testing, a probability lower than 0.05 was considered statistically significant. No adjustment for multiplicity was applied. All statistical tests were two-sided.

Analyses were conducted using SAS v9.4 (SAS Institute, Cary, NC, USA), Excel 365 (Microsoft, Redmond, WA, USA), SPSS for Windows v15.0 (IBM, Armonk, NY, USA), and R v4.1.3 (R Foundation, Kaysville, UT, USA).

## 3. Results

**Figure 2:**
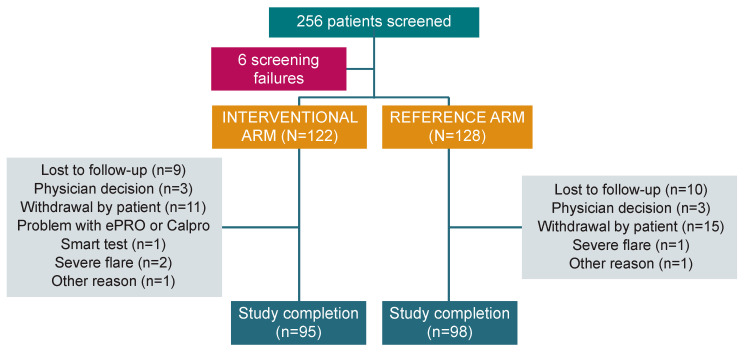
Study flow chart. PRO: Patient-reported outcome.

In the period between October 2019 and November 2022, 256 patients from 31 institutions across six European countries were screened, and 250 patients (median age 40.0 [18–78] years; 54.0% male) were enrolled in the study: 122 in the interventional arm and 128 in the reference arm. After randomisation, 193 patients completed the study (77.2%): 95 (77.9%) in the interventional arm and 98 (76.6%) in the reference arm (Figure 2). The mITT population included 241 patients (118 in the interventional arm and 123 in the reference arm), while the PP analysis included 129 patients (52 and 77, respectively).

### 3.1. mITT Analysis

**Figure 3:**
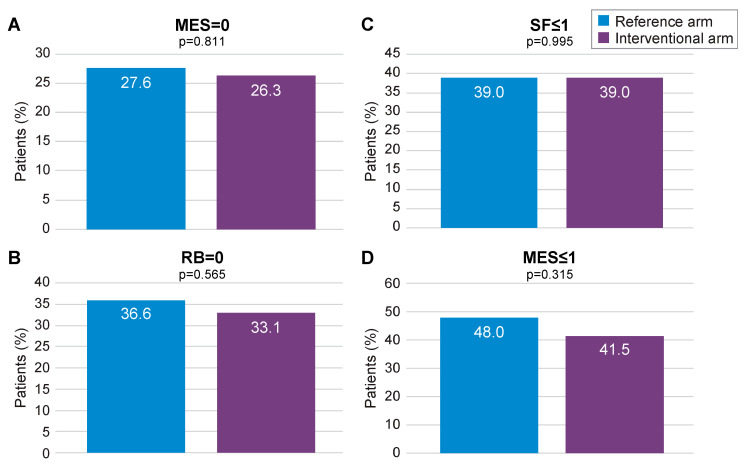
Primary (MES = 0) and secondary remission endpoints (mITT population). MES: Mayo Endoscopic Subscore; mITT: modified intention-to-treat; RB: rectal bleeding; SF: stool frequency. (**A**) proportion of patients with MES = 0 in the reference arm and in the interventional arm; (**B**) proportion of patients with SF < or =1 in the reference arm and in the interventional arm; (**C**) proportion of patients with RB=0 in the reference arm and in the interventional arm; (**D**) proportion of patients with MES < or =1 in the reference arm and in the interventional arm.

At 12 months, MES = 0 (primary outcome) was achieved by 26.3% of patients in the interventional arm and by 27.6% in the reference arm (*p* = 0.811; Figure 3A). Absence of RB was achieved by 33.1% of patients in the interventional arm versus 36.6% in the reference arm (*p* = 0.565; Figure 3B), whilst normal SF was reported at 39.0% in both arms (*p* = 0.995; Figure 3C). Endoscopic remission, defined as a MES ≤ 1, was achieved by 41.5% of patients in the interventional arm and 48.0% in the reference arm (*p* = 0.315; Figure 3D). In both arms, patients showed a similar level of improvement in their quality of life and reduction of work impairment through month 12 (Supplementary Figures S1 and S2). FC levels were found to decrease over time in the interventional arm (Supplementary Figure S3). The median number of FC dosages per patient was 7.5. Dose escalation was significantly more frequent in the interventional arm than in the reference arm (22.9% vs. 8.1%; *p* = 0.001). Combined steroid-free clinical and endoscopic remission (MES = 1, RB = 0 and SF ≤ 1) was achieved in 9.9% in the interventional arm compared to 5.6% in the reference arm (*p* = 0.405), whereas steroid-free clinical remission (RB = 0 and SF ≤ 1) was achieved in 22.9% in the interventional arm versus 25.2% in the reference arm (*p* = 0.763).

### 3.2. PP Analysis

In the PP analysis, the primary outcome was achieved by 36.5% in the interventional arm versus 28.6% in the reference arm (*p* = 0.340; Supplementary Figure S4A). Absence of RB was reported by 40.4% of patients in the interventional arm and 42.9% in the reference arm (*p* = 0.780; Supplementary Figure S4B), whereas normal SF was observed in 48.1% in both arms (*p* = 0.998; Supplementary Figure S4C). Endoscopic remission (MES ≤ 1) at month 12 was achieved by 57.7% of patients in the interventional arm versus 50.6% in the reference arm (*p* = 0.431, Supplementary Figure S4D). No differences were observed in both quality-of-life improvement and reduction in work productivity through month 12 (Supplementary Figures S5 and S6). The PP analysis confirmed reduction of FC through month 12 in the interventional arm (Supplementary Figure S7). Combined steroid-free clinical and endoscopic remission (MES = 1, RB = 0 and SF ≤ 1) was achieved in 17.1% in the interventional arm compared with 8.5% in the reference arm (*p* = 0.312), whereas steroid-free clinical remission (RB = 0 and SF ≤ 1) was achieved by 38.9% in the interventional arm versus 46.8% in the reference arm (*p* = 0.509).

#### Complementary Analyses

**Figure 4:**
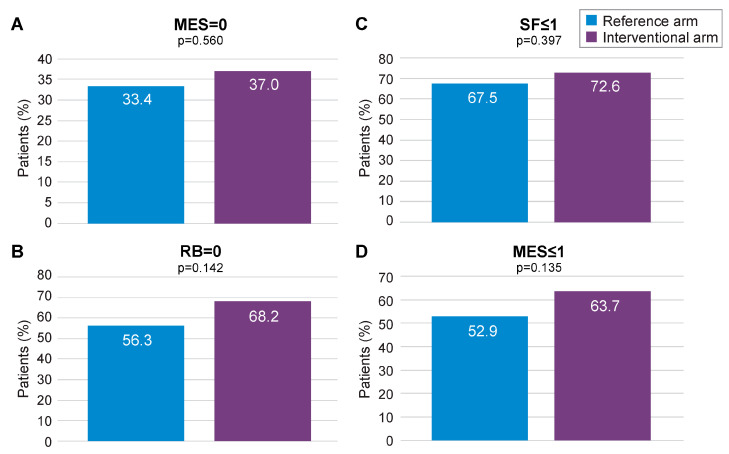
Primary (MES = 0) and secondary remission endpoints for the mITT population following MCMC imputation. MES: Mayo Endoscopic Subscore; MCMC: Monte Carlo Markov Chain; mITT: modified intention-to-treat; RB: rectal bleeding; SF: stool frequency. (**A**) proportion of patients with MES = 0 in the reference arm and in the interventional arm; (**B**) proportion of patients with SF < or =1 in the reference arm and in the interventional arm; (**C**) proportion of patients with RB = 0 in the reference arm and in the interventional arm; (**D**) proportion of patients with MES < or =1 in the reference arm and in the interventional arm.

**Figure 5:**
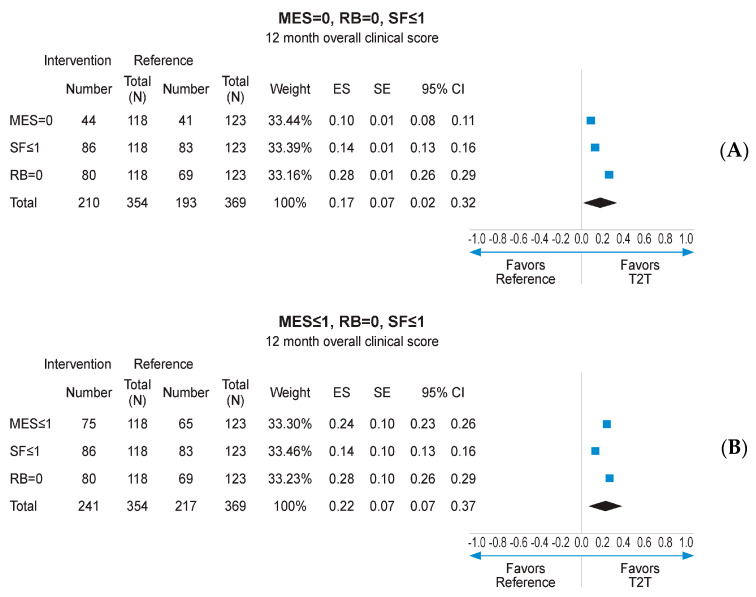
Meta-analysis of individual endpoints following MCMC imputation of mITT population. CI: confidence interval; ES: effect size; MES: Mayo Endoscopic Subscore; MCMCL Monte Carlo Markov Chain; mITT: modified intention-to-treat; RB: rctal bleeding; SF: stool frequency. (**A**) meta-analysis of individual patients achieving MES = 0, RB = 0, SF < or =1; (**B**) meta-analysis of individual patients achieving MES < or =0, RB = 0, SF < or =1.

Missing data imputation with the MCMC method allowed to compare the outcomes of interest on the whole mITT population (118 in the interventional arm and 123 in the reference arm), with MCMC imputation providing 99 imputed datasets. Subsequent reanalysis of the primary endpoint (MES = 0) found a numerical advantage for the interventional arm over the reference arm (37.0% vs. 33.4%, respectively; Figure 4A). The secondary endpoints of RB = 0, SF ≤ 1, and MES ≤ 1 at 12 months also displayed numerical advantage for the interventional arm following MCMC imputation (Figure 4A–D). Logistic regression analysis pooling results for MES, SF, and RB at 12 months, using data derived from MCMC imputation, found a statistically significant advantage for the interventional arm over the reference arm (*p* < 0.001; Supplementary Figure S8). When combined in a fixed effects meta-analysis, the composite endpoint of MES = 0, RB = 0, and SF ≤ 1 at 12 months was achieved at a significantly higher rate in the interventional arm than the reference arm (ES: 0.17, 95% CI 0.02–0.32; *p* < 0.05; Figure 5A). A similar result was obtained for MES ≤ 1, RB = 0, and SF ≤ 1 at 12 months (ES: 0.22; 95% CI 0.07–0.37; *p* < 0.05; Figure 5B).

## 4. Discussion

To the best of our knowledge, OPTIMISE is the first study to assess the benefits of a T2T approach in patients with mild-to-moderate UC. Other strengths are the multicenter and randomised study design and a well-established methodology, which reduce the risk of bias. Being a pragmatic trial, the study was conducted in a real-world population of patients with UC at potential high risk of non-compliance to medications and procedures [23]; moreover, the enrolment period overlapped with the COVID-19 pandemic, resulting in a high rate of dropouts based upon physicians’ (33.0%) or patients’ decisions (45.6%). These main limitations reduced the originally calculated sample size from 250 to 193 patients as well as causing a high percentage of missing data, resulting in increased imprecision in the results and may have contributed to the lack of a significant difference between the two management approaches in the primary analysis. The high dropout rate related to the COVID-19 pandemic might affect the generalisability of our findings. However, it was supposed to be overcome by statistical methods. Indeed, after adjusting for missing data to enable a more precise estimation of the difference between the two approaches, it was found that targeting normalisation of FC was associated with a significantly higher probability of achieving combined clinical and endoscopic remission at month 12 (17–22% absolute benefit over reference arm). There was also a numerically higher difference in favour of the T2T strategy compared to the reference arm for the individual primary and secondary outcomes, although these differences were not statistically significant. Importantly, for patients included in the interventional arm, median FC values dropped from baseline through month 12 as a result of treatment escalation based on this biomarker. Another limitation is the open-label design, which may introduce biases related to the participants’ and investigators’ awareness of the treatment allocation.

The results of the OPTIMISE study are supported by a recently conducted cost-effectiveness analysis based on a theoretical comparison between the two strategies [24]. The economic model showed that targeting FC increased the time spent by a patient in clinical remission with FC ≤ 100 μg/g (approximately 2 months) and decreased the number of relapses (−20.9%) *per* patient. The STARDUST and CALM trials enrolled patients with CD and provided contrasting results on the effectiveness of a T2T approach through 48–52 weeks of follow-up [22,25]. The CALM trial [17] supported the use of tight monitoring of biomarkers, with the primary endpoint of mucosal healing (CD Endoscopic Index of Severity < 4) achieved in 46% of patients compared with 30% for those whose disease was managed based on symptoms alone (*p* = 0.010). [22]. The STARDUST trial, however, reported a similar result to that observed in the OPTIMISE study (following MCMC imputation), with only a numerical advantage for the primary endpoint of endoscopic response (Simple Endoscopic Score in CD ≥ 50% decrease from baseline) in patients following a T2T rather than symptom-based approach (38% vs. 30%, respectively; *p* = 0.087) [25]. The results from these two trials, as well as those from OPTIMISE, highlight the challenges of undertaking T2T studies in IBD, particularly when undertaken in a real-world, clinical practice setting.

It is important to note that the OPTIMISE study was designed to consider only 12 months of follow-up. Recent data suggest that targeting disease clearance (defined as a combination of clinical, biomarker, endoscopic, and histological remission) results in a reduced risk of hospitalisation and surgery over a median period of 24 months [26]. Another large cohort study suggested that a T2T approach results in a similarly low incidence of IBD-related cancer to that seen in the general population and can aid in the detection of malignancies at an early stage [27]. The long-term effectiveness of a T2T approach in UC, particularly in patients with a mild-to-moderate disease course with fewer relapses (measurable events) than those with moderate-to-severe disease, is therefore worthy of further investigation.

## 5. Conclusions

Our results provide real-world evidence of the effectiveness of a T2T approach based on FC in patients with mild-to-moderate UC. Compared to a symptom-based approach, T2T was associated with higher numbers of patients in combined clinical and endoscopic remission. Further longer-term studies are needed to confirm our findings.

## Figures and Tables

**Table 1:** Treatment algorithm.

Site of Disease	Prior Relapse (Screening)	Escalation Steps (Active Disease/Relapse)			De-Escalation Steps (Remission)	
		1st Step (Baseline)	2nd Step	3rd Step	1st Step	2nd Step/ Maintenance
Proctitis UC	Oral 5-ASA 2.4 g/day or no treatment	5-ASA suppository ≥ 1 g/day + oral 5-ASA ≥ 2.4 g/day	Rescue Therapy **	Rescue Therapy **	Oral 5-ASA ≥ 2.4 g/day	Oral 5-ASA < 2.4 g/day
Left-sided UC		5-ASA enema ≥ 1 g/day + oral 5-ASA ≥ 2.4 g/day	2nd generation corticosteroids *			
Extensive UC						

* Second-generation corticosteroids (e.g., with a colonic release mechanism and low systemic bioavailability), including oral beclomethasone dipropionate (5 mg/day) or budesonide MMX (9 mg/day). ** At the discretion of the physician, including systemic corticosteroids, immunosuppressants, and/or biologics. 5-ASA: 5-aminosalicylic acid; UC: ulcerative colitis.

## Data Availability

Data is available on request due to privacy and ethical restrictions.

## Supplementary Materials

The following supporting information can be downloaded at: https://www.mdpi.com/article/10.3390/jcm13175147/s1, Supplementary Figure S1. SIBDQ Global Score: mean value (SD) over time (mITT population); Supplementary Figure S2. WPAI Overall Work Impairment: mean value (SD) over time (mITT population); Supplementary Figure S3. Faecal calprotectin (FC): mean levels over time (mITT population): Supplementary Figure S4. Primary (MES = 0) and secondary remission endpoints (PP analysis): Supplementary Figure S5. SIBDQ Global Score: mean value (SD) over time (PP analysis); Supplementary Figure S6. WPAI Overall Work Impairment: mean value (SD) over time (PP analysis); Supplementary Figure S7. Faecal calprotectin (FC): mean levels over time (PP analysis); Supplementary Figure S8. Receiver operating characteristic (ROC) curve analysis against planned treatment*. Appendix S1. List of all principal investigators who participated in the OPTIMISE study.
