# Supplementary material for: Clinical Trial: A Pragmatic Randomised Controlled Study to Assess the Effectiveness of Two Patient Management Strategies in Mild to Moderate Ulcerative Colitis—The OPTIMISE Study
Source: J Clin Med. 2024 Aug 30;13(17):5147. doi: 10.3390/jcm13175147 (PMC11395821; doi:10.3390/jcm13175147)
Supplement: Supplementary file 1 [file jcm-13-05147-s001.zip › Appendix S1.pdf]

Appendix S1. List of all principal investigators who participated in the OPTIMISE study

Country	Institution	Principal Investigator
Czech Republic	Hepato-Gastroenterologie HK, s.r.o.	Vanasek, Tomas
Czech Republic	ISCARE a.s.	Lukas, Milan
Czech Republic	KZ a.s.-Masaryk Hospital	Tichy, Michal
Czech Republic	Vojenska nemocnice Brno p.o.	Stepek, David
Czech Republic	Mediendo s.r.o.	Suchanek, Stepan
Hungary	Bacs-Kiskun Megyei Oktatokorhaz, a Szegedi Tudomanyegyetem Oktatokorhaza	Dubravcsik, Zsolt
Hungary	Pannonia Mgánorvosi Centrum Kft	Schnabel, Robert
Hungary	Szegedi Tudomnyegyetem	Molnar, Tamas
Hungary	Semmelweis Egyetem, Isz. Sebeszeti es Intervencios Gasztroenterologiai Klinika	Miheller, Pal
Italy	Azienda Ospedaliero-Universitaria Pisana	Costa, Francesco
Italy	ASST-FBF Luigi Sacco Hospital	Maconi, Giovanni
Italy	IRCCS Ospedale San Raffaele	D'Amico, Ferdinando
Netherlands	Franciscus Gasthuis & Vlietland	West, Rachel
Netherlands	Jeroen Bosch Ziekenhuis	Nissen, Loes
Netherlands	Catharina Hospital	Gilissen, Lennard
Poland	SANTA FAMILIA Centrum Badan, Profilaktyki i Leczenia	Wisniewska-Jarosinska, Maria
Poland	Centrum Medyczne LukaMed	Drobinski, Piotr
Poland	Melita Medical	Fronik, Grzegorz
Poland	Karkonoskie Centrum Bada Klinicznych – Lexmedica SP Z O.O	Fic, Miroslaw
Poland	NZOZ Vitamed	Walczak, Michal
Poland	H-T. Centrum Medyczne sp. z o.o.	Romanczyk, Tomasz
Poland	Centrum Diagnostyczno Lecznicze Barska	Kowalski, Maciej
Poland	Szpital Uniwersytecki Nr2 im. Dr J.Biziela	Manerowski, Marcin
Poland	NZOZ Centrum Gastroenterologii w Wodzislawiu Slaskim	Skupien, Stanislaw
Poland	Korczowski Bartosz, Gabinet Lekarski	Korczowski, Bartosz
Poland	Osrodek Badan Klinicznych Clinsante	Wiatr, Michal
Poland	Centrum Medyczne Medyk	Filip, Rafal
Poland	Saint Barbara's Regional Specialist Hospital No. 5 in Sosnowie	Duda-Raszewska, Barbara
Slovakia	ENDOMED s.r.o.	Fedurco, Miroslav
Slovakia	Gastro LM s.r.o.	Mihalkanin, Lubomir

Slovakia	Pigeas s.r.o.	Balaz, Dusan
