# Supplementary material for: Clinical Trial: A Pragmatic Randomised Controlled Study to Assess the Effectiveness of Two Patient Management Strategies in Mild to Moderate Ulcerative Colitis—The OPTIMISE Study
Source: J Clin Med. 2024 Aug 30;13(17):5147. doi: 10.3390/jcm13175147 (PMC11395821; doi:10.3390/jcm13175147)
Supplement: Supplementary file 1 [file jcm-13-05147-s001.zip › Supplementary Figure S1.pdf]

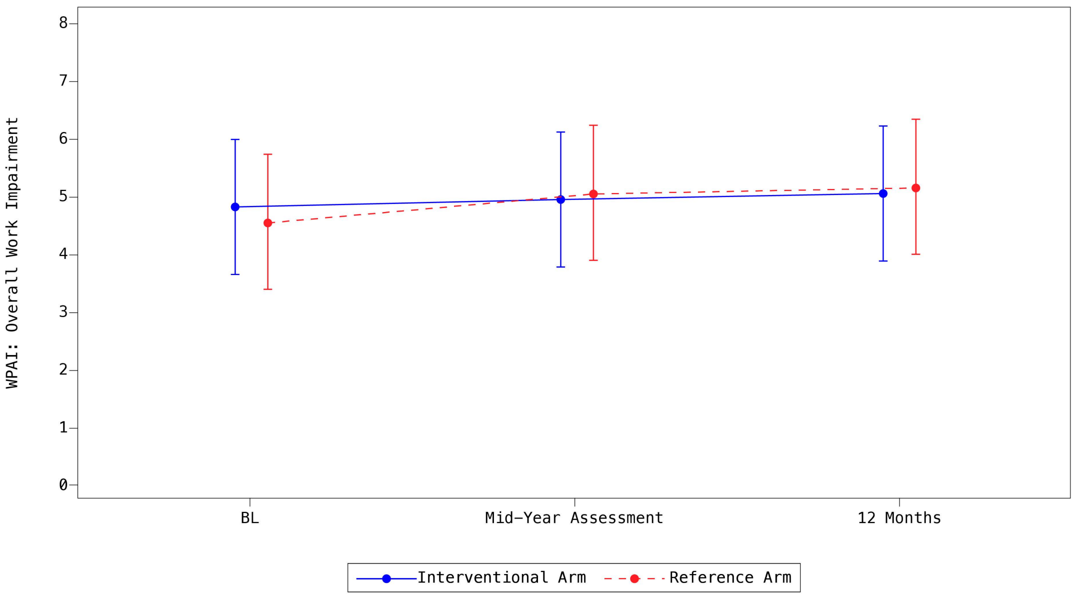

BL: baseline; mITT: modified intention-to-treat; SD: standard deviation; SIBDQ: Short Inflammatory Bowel Disease Questionnaire

For Short Form-36 (SF-36) there was no significant difference between arms regarding the values of SF-36 Scores or their changes from baseline at the different times of the study across the different scales. Significant within-patient changes between baseline and 12 months were found for all scales for both arms with the exception of: Physical Functioning Norm-Based Score, which did not exhibit significant changes for both arms; General Health Norm-Based Score, which increased significantly in the reference arm but not in the interventional arm; and, Mental Health Enhanced Score that decreased significantly in reference arm but was at the limit of the significance (p=0.053) in the intervention arm.
