# Supplementary material for: Clinical Trial: A Pragmatic Randomised Controlled Study to Assess the Effectiveness of Two Patient Management Strategies in Mild to Moderate Ulcerative Colitis—The OPTIMISE Study
Source: J Clin Med. 2024 Aug 30;13(17):5147. doi: 10.3390/jcm13175147 (PMC11395821; doi:10.3390/jcm13175147)
Supplement: Supplementary file 1 [file jcm-13-05147-s001.zip › Supplementary Figure S2.pdf]

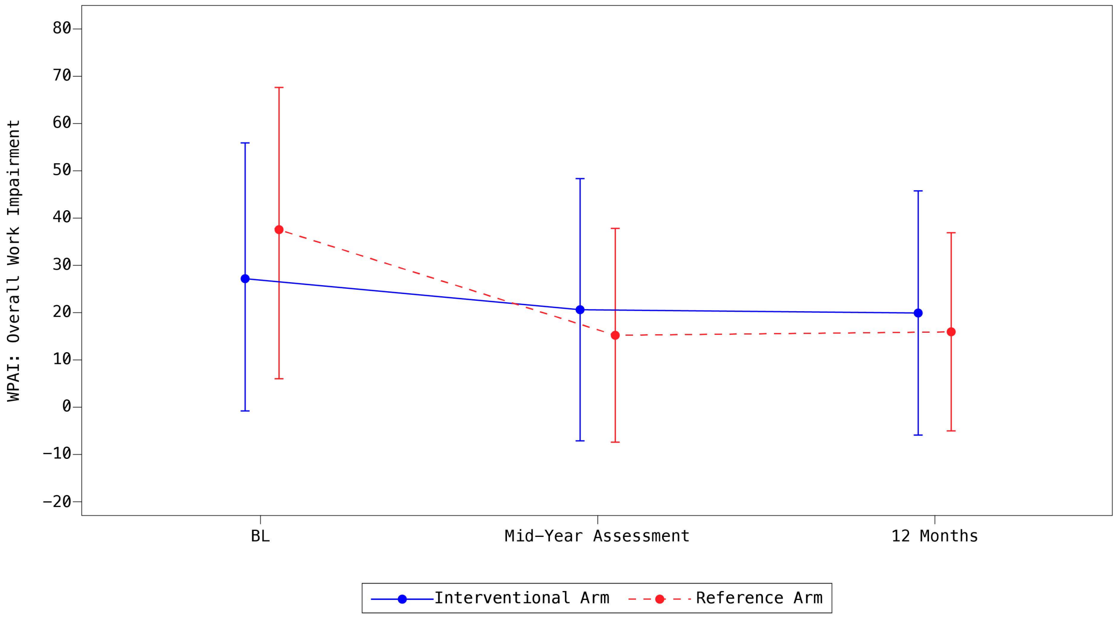

BL: baseline; mITT: modified intention-to-treat; SD: standard deviation; WPAI: Work Productivity and Activity Impairment
