# Supplementary material for: Clinical Trial: A Pragmatic Randomised Controlled Study to Assess the Effectiveness of Two Patient Management Strategies in Mild to Moderate Ulcerative Colitis—The OPTIMISE Study
Source: J Clin Med. 2024 Aug 30;13(17):5147. doi: 10.3390/jcm13175147 (PMC11395821; doi:10.3390/jcm13175147)
Supplement: Supplementary file 1 [file jcm-13-05147-s001.zip › Supplementary Figure S8.pdf]

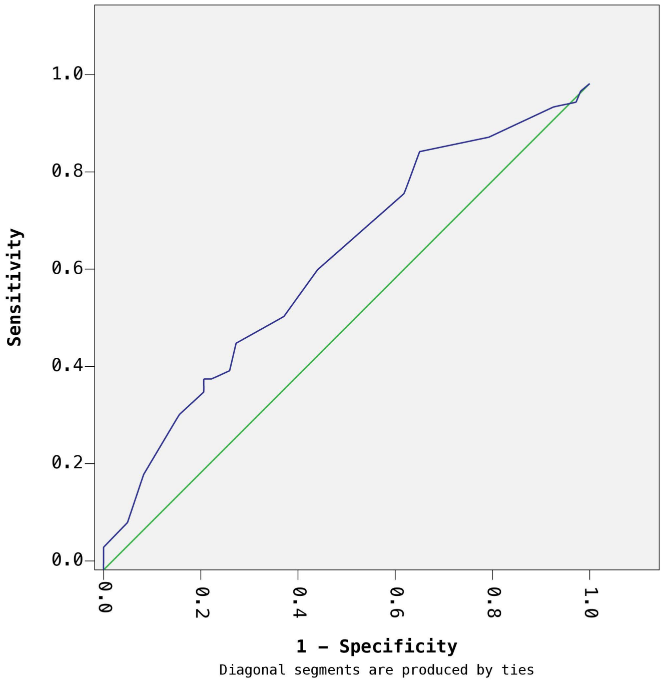

Area Under the Cuve

Test Results Variable(s): Predicted probability

Area	Std. Error[a]	Asymptotic Sig.[b]	Asymptotic 95% Confidence Interval	
			Lower Bound	Upper Bound
.632	.035	.000	.564	.701

*Logistic regression analysis was performed by mapping the MCMC results to a theoretical population of 250 patients, with planned treatment (interventional arm or reference arm) used as the dependent variable and 12-month results for MES, SF and RB used as the predictor variables. The derived predictor function was then used for ROC curve analysis.
MCMC: Monte Carlo Markov Chain (imputation)
