# Supplementary figures and images for: Clinical Trial: A Pragmatic Randomised Controlled Study to Assess the Effectiveness of Two Patient Management Strategies in Mild to Moderate Ulcerative Colitis—The OPTIMISE Study
Source: J Clin Med. 2024 Aug 30;13(17):5147. doi: 10.3390/jcm13175147 (PMC11395821; doi:10.3390/jcm13175147)

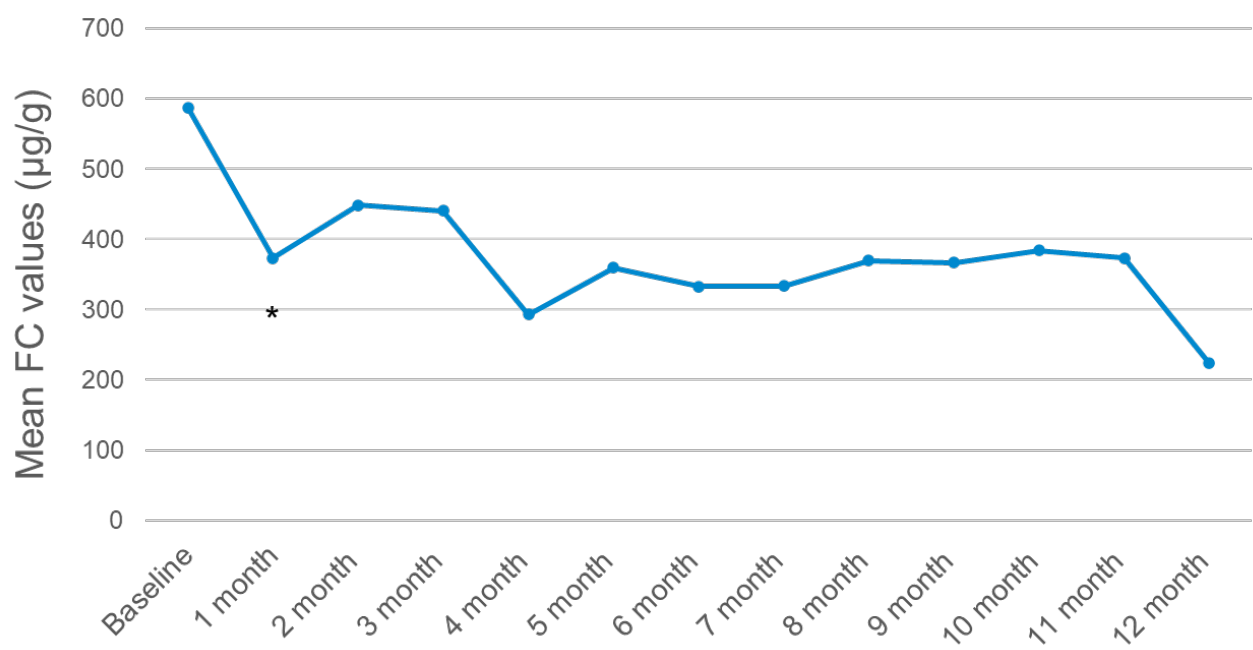

\*p=0.011 vs baseline (other timepoints not significant)

mITT: modified intention-to-treat

Supplement: Supplementary file 1 [file jcm-13-05147-s001.zip › Supplementary Figure S3.pdf]

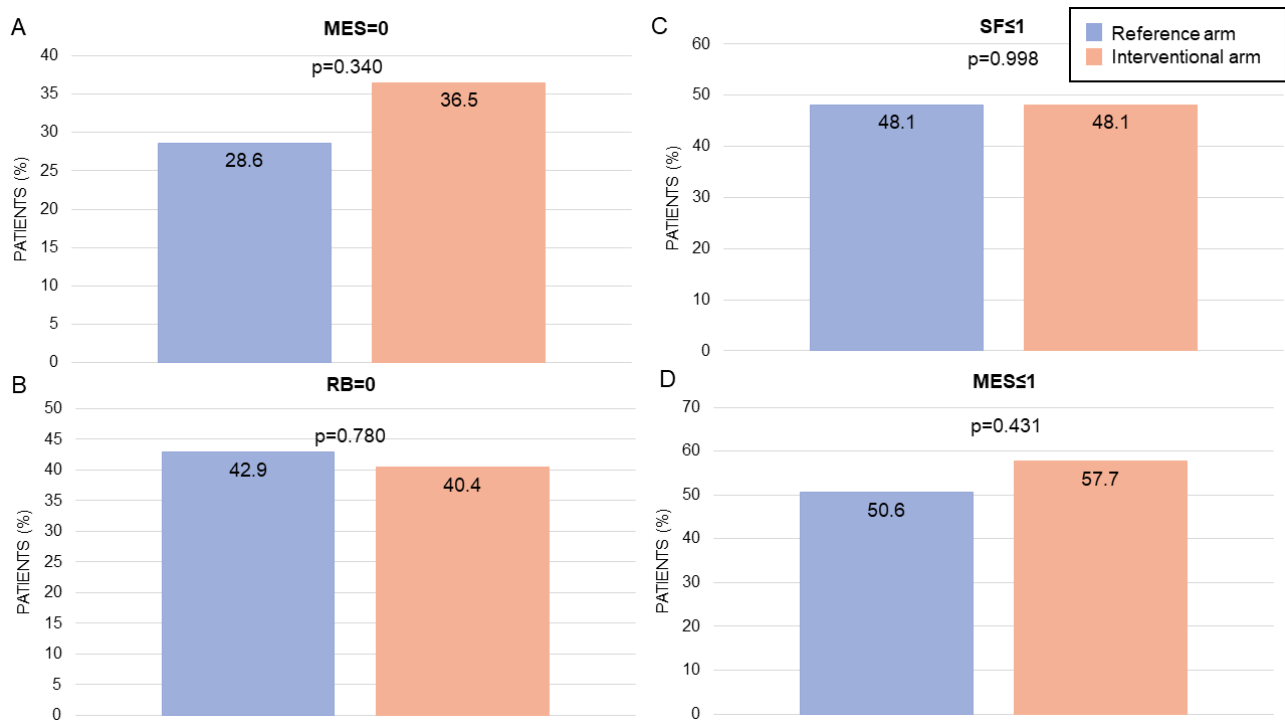

MES: Mayo Endoscopic Subscore; PP: per-protocol; RB: rectal bleeding; SF: stool frequency

Supplement: Supplementary file 1 [file jcm-13-05147-s001.zip › Supplementary Figure S4.pdf]

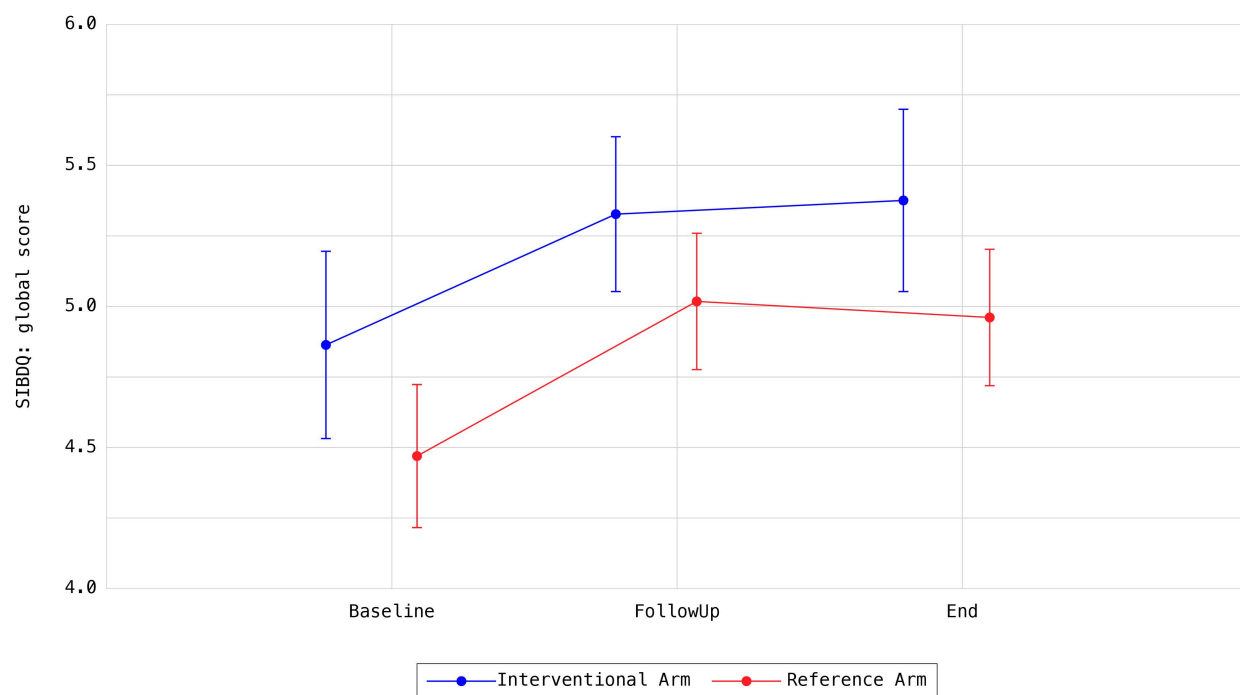

PP: per-protocol; SD: standard deviation; SIBDQ: Short Inflammatory Bowel Disease Questionnaire

Supplement: Supplementary file 1 [file jcm-13-05147-s001.zip › Supplementary Figure S5.pdf]

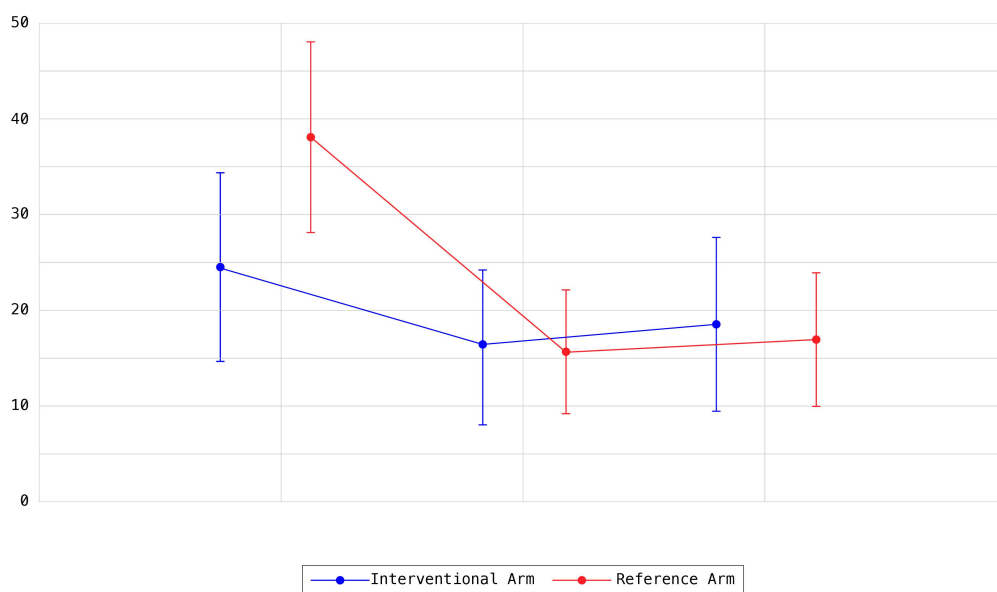

PP: per-protocol; SD: standard deviation; WPAI: Work Productivity and Activity Impairment

Supplement: Supplementary file 1 [file jcm-13-05147-s001.zip › Supplementary Figure S6.pdf]

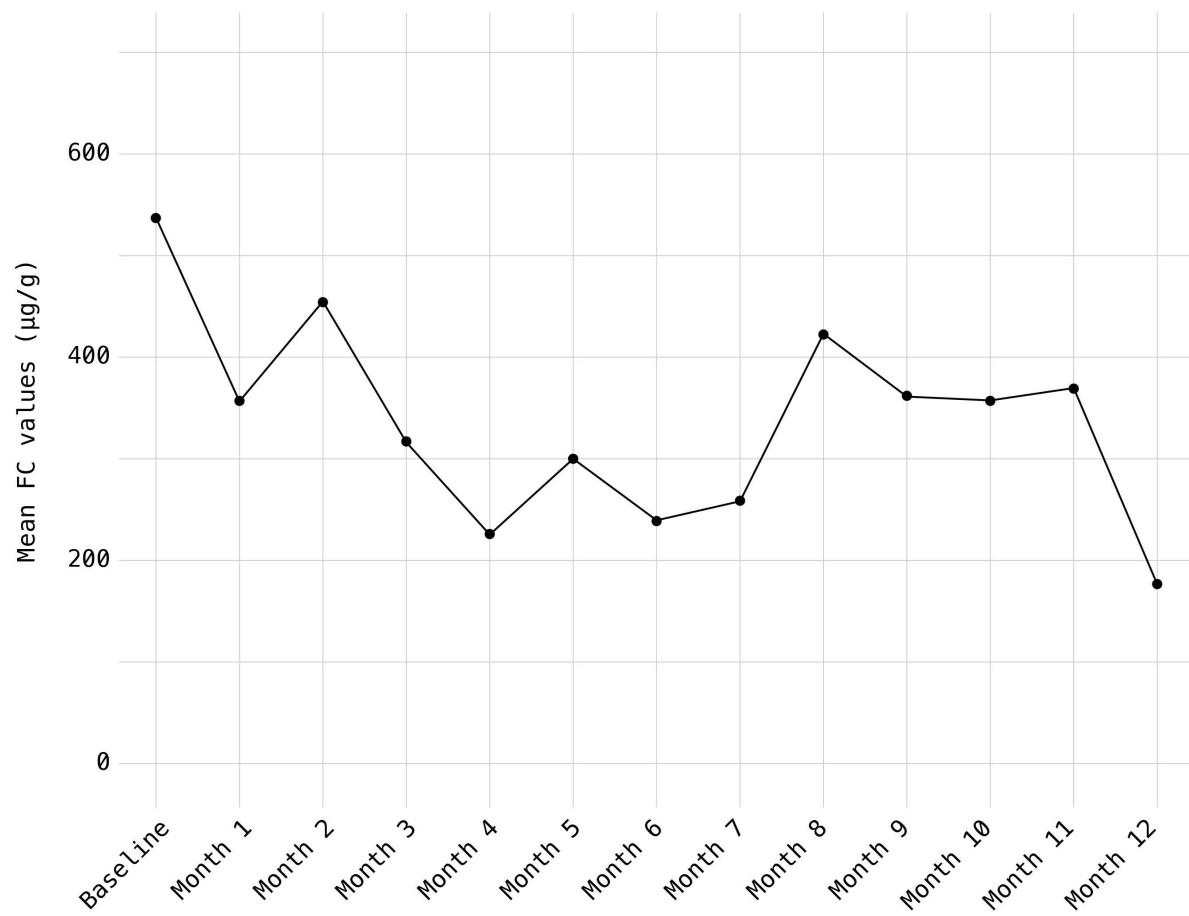

PP: per-protocol

Supplement: Supplementary file 1 [file jcm-13-05147-s001.zip › Supplementary Figure S7.pdf]
